# Bibliographical Notices

**Published:** 1851-05

**Authors:** 


### BIBLIOGRAPHICAL NOTICES.

*The Pharmacopœia of the United States, by authority of the National Medical Convention, held at Washington A. D.* 1851. Philadelphia; Lippincott, Grambo & Co., 1851.

This is the fourth issue of our National Pharmacopœia, promulgated on the highest medical authority which exists in this country, a convention formed by representatives, consisting of physicians and pharmaceutists, from all sections of its vast domain. Every portion of the Union is entitled to representation in the formation of this work, and the fault of failure to take part in it lies solely at the door of the medical corporations. The present edition is the third revision.

The history of the Pharmacopœia is, perhaps, familiar only to those who are conversant with the progress of Pharmacy in the United States. More than thirty years ago the necessity of such a work became apparent, and it was determined, by public spirited men of high scientific acquirements, to accomplish it. The difficulties which were encountered and overcome, constitute a part of the records of the time, and may be learned from the medical periodicals then issued. Each succeeding edition has to a greater extent confirmed the ideas entertained of its necessity and importance.

From an examination of the present edition, it is evident that careful labor has been expended, to make it accord with the great progress in medical and pharmaceutic research, which has characterized the last decennial period.

After an historical introduction, which details merely the formation of the Convention, and the mode in which the revision was effected, we are presented with a preface, containing information highly interesting. We refer to that which pertains to the plan which has been adopted, and to an exposition of the general principles on which it has been founded.

The older members of the profession may recollect that the original work was published in the Latin tongue, that the subsequent edition was in Latin and English, and the next in English only. The present revision is as the last, in English. Much may be said upon both sides of the question, was it expedient to drop the Latinity? In the ancient language of science more of learning and dignity is displayed undoubtedly, but this is lost in the necessary accommodation of expression to the new discoveries and processes which require exposition. Pure classical Latin, the Latin of Celsus, cannot accommodate itself to the hundred requisitions of speech not dreamed of in his philosophy, and such Latin as can at the present time be framed, is a caricature on ancient learning.

The argument has been used, that the Latin is the language of science. It was at one time the language of the learned, but at a time when other languages were in formation, when there existed a necessity for a general medium; the necessity does not exist at present. More, now, can read English than Latin, and write it also, which was not the case when the latter was in vogue. The object of using any language is general utility, and this, with the reasons given, was, with the framers of the Pharmacopœia, conceived to be ample justification for the introduction of our own vernacular. They might have cited precedents in the Codex, the Edinburgh Pharmacopœia, and the late edition of the Dublin.

What is true with respect to common familiar directions for the formation of preparations, is not so with respect to nomenclature. A classical foundation is here most productive of what a nomenclature should be, "simple, expressive, distinctive and convenient." The pages which are devoted to an exposition of the principles adopted in the formation of the nomenclature, are full and explanatory. They should not be perused, but studied.

From the great fulness of the original list, an opportunity has been given of introducing but few additional medicines.

Those added have been cautiously admitted, and consist of such as have, by strong testimony, claims to admission. We notice seventeen such additions. The advance of science and discovery, however, has entailed greater amplification with respect to preparations, and fifty-three new ones have been accordingly introduced ; while the medicines dismissed consist of three, and the preparations are two in number. How much more the work embraces than the former issue, may be judged of from the foregoing statements.

Among the articles introduced, we find Aconite Root, Extract of Hemp, Cod Liver Oil, Citrate of Potassa, Nitrate of Lead, as well as some articles long used, but heretofore not having a place in our Pharmacopœia. Upon turning over the list of the materia medica, it will be found that changes have been made in nomenclature, of which it is of consequence that physicians should be aware ; thus, for Myroxylon and Tolu, in the former editions, are substituted Balsamum Peruvianum, and Balsamum Tolutanum. This became necessary from the change in the name of the plants affording the articles, which are now regarded as species of Myrospermum. Brominium and Iodinium are here found instead of Brominum and Iodinum, a change made to render the nomenclature uniform with that of the British Colleges. Many other changes of the kind are met with, and in order to avoid a misunderstanding, the old names are placed in brackets alongside of the new. The botanical names appear to have been made generally to conform with the advance of this science, hence the substitution of different generic and specific appellations for some to which the ear had been long accustomed; thus Assafœtida is referred to the Narthex Assafœtida, Colocynth to the Citrullus Colocynthis, Camphor to Camphora Officinarum, Cinnamon to the Cinnamomum Zeylanicum and Aromaticum. The name of only one chemical, so far as we have discovered, is thus changed, and that is Zinci Carbonas to Calamina.

A difficulty has always existed with respect to the strength of nitric acid, which could with difficulty be procured of the old standard, sp. gr. 1.5. To accommodate the chemical manufacturer, the specific gravity of 1.42 has been authorized. A chemical sanction for this is given by Mr. Graham, who states that an acid of the strength adopted, is the "proper nitrate of water; and of the four atoms of water which it contains, one is combined with the acid as a base, and may be termed basic water, while the other three are in combination with the nitrate of water, and may be termed the constitutional water of that salt."

The introduced preparations are so numerous, that we have not space to enumerate them; a few selected ones will answer our purpose. The Ferri Pulvis occupies an important position, at the present time, among the preparations of iron; this is the reduced oxide of iron by hydrogen, which is sometimes known as *Quevenne's iron*, for an abundant domestic supply of which we are much indebted to Prof. Procter. It is the most effective of the preparations of pure iron. Chloroform, Collodium, Glycerina, Zinci Carbonas Præcipitatus, Arsenici Iodidum are others most prominent.

There is a class of preparations which are entirely new in a Pharmacopœia, that of fluid extracts. These preparations are composed of substances whose active principles can be isolated by the proper solvents, and a concentration of them effected, so as to administer the proper dose in the least bulk. Such are the Fluid Extract of Rhubarb, Fluid Extract of Senna, &c., which are, notwithstanding their elaborate preparation, efficient medicines.

We may, in conclusion, remark, that the edition of 1851 is the most elegant of all the editions which have been given to the public. In paper and typography it far exceeds the issue of 1842; independently of its scientific merit, in this respect, it is a most creditable addition to our medical literature. Our only regret is, that the high price at which it is placed will serve to keep it out of the hands of the profession, rather than to facilitate its introduction where it is most desirable it should be in daily use.

*On the Diseases of Menstruation and Ovarian Inflammation, in connection with Sterility, Pelvic Tumors and Affections of the Womb.* By EDWARD JOHN TILT, M. D., Physician to the Farrington General Dispensary, and to the Paddington Free Dispensary for the Diseases of Women and Children. "*Omne animal ab ovo.*" New York: J. S. & W. Wood, 1851.

The object of the work of Dr. Tilt is to show how inflammation, by reacting on the ovaries, produces diseases of menstruation, sterility and uterine disease; and although we are, for the most part, inclined to look with suspicion upon the productions of of hobby-riders, we are free to confess, that we have risen from the perusal of this little volume with a deep sense of the indebtedness of the profession to its author for the aid that he has afforded them in clearing away much of the obscurity of uterine pathology.

The work opens with six preliminary questions, followed by a prolegomenon in which the subject of ovarian inflammation is introduced; the confusion relative to its history discussed; its frequency proved by that of inflammatory morbid tumors in the ovaries; the reason for the past neglect of ovarian pathology; the ignorance of ovarian physiology, and the pre-eminence traditionally assigned to the uterus among the organs of reproduction; the difficulty of exploring organs so small and so deeply seated; the similitude of many of the symptoms of sub-acute ovaritis to those of inflammation of the womb; the popular conviction that menstruation is a natural function, and that there is no remedy for whatever evils may attend it; the repugnance of patients to submit to the necessary modes of investigation; the repugnance of physicians to press for it when not imperatively demanded; all of which subjects are treated in a lucid manner by the author. After this comes a sketch of ovarian bibliography, a section on the different ovarian explorations:—by the abdomen, the vagina, the rectum, and the double touch.

The body of the work is divided into nine chapters, of which the first five are occupied in the discussion of sub-acute ovaritis, its causes, symptoms, terminations and treatment; whilst the last four are devoted to acute ovaritis, its causes, diagnosis terminations and treatment. A few extracts will better enable our readers to understand the style and mode of treating the subject, by our author, than any written description of our own.

"SUB-ACUTE OVARITIS.

*Syn.*—Chronic ovaritis; secondary pelvic inflammation. (Dr. Kennedy.)—Abdominal inflammation.—Menstrual colics.—Amenorrhœa.—Dysmenorrhêe hystêralgique. (Gêndrin.)—Dysmenorrhœa.—Menorrhagia.—Hysteria.

*Def.*—Swelling of the ovaria, with increase of heat, and pain upon pressure, accompanied by intermittent or permanent pain or uneasiness in the ovarian region, radiating to the loins and thighs, and producing, according to the constitution of the patient, an arrest of menstruation, or its profuse flow, intense local pain, or hysterical symptoms.

By *sub-acute* inflammation, as distinguished from *acute*, we do not so much imply a difference in the intrinsic nature of the morbid phenomena, as a limitation of the inflammatory action to certain distinct parts of the ovaries, as the ovarian follicle, and to portions of the ovarian tissue so small that they give rise to little swelling, and to no febrile action; and here we may point out, as peculiar properties of the sexual system in women, the liability to inflammation of certain portions of the generative apparatus, in which the others may not participate—a peculiarity to which the ovary is still more liable, on account of its complex structure.

Sub-acute ovaritis, whether primarily developed as such, or supervening on the acute inflammation of the ovaries, is necessarily a chronic disease, from the circumstance of the ovaries being subject to a periodical augmentation of nervous and sanguineous excitement. Chronic ovaritis is always sub-acute; and as sub-acute inflammation of the ovaria is often present without being chronic, we have thought it best to adopt the appellation common to them both, instead of that generally made use of. Sub-acute ovaritis is by far the more common, and, therefore, we will first proceed to its investigation.

It is evident, however, that in the determination of causes, in the symptoms, and in the treatment of these two diseases, we shall find a great similarity, we shall also find that they may pass the one into the other, the sub-acute being exasperated into the acute, while acute ovaritis sometimes becomes sub-acute, or chronic, as it is then generally termed.

We admit, then, two forms of ovaritis—1st, the sub-acute; 2nd, acute ovaritis; and, in attempting for the ovaries what has been so felicitously done for other organs, we will endeavour to show that the groups of symptoms associated under the classic names of amenorrhœa, dysmenorrhœa, menorrhagia, and hysteria, are often the mere symptoms of sub-acute ovaritis.

We stand not alone in this belief. Joseph Frank and Dr. Chester hold the same creed. Dr. Robert Lee is much of the same opinion. Clarus distinctly says, that he considers the disorders of menstruation as the symptoms of chronic ovaritis; and Dr. Rigby strenuously advocates the same doctrine."

Again, in describing the symptoms of sub-acute ovaritis, the author gives the following clear account:

The patient experiences a dull pain in the ovarian region, often imperceptible when she is in a state of repose, but brought on by walking, riding, by any sudden movement, or even by pressure on the side. The pain is also increased by the act of straightening the thigh upon the pelvis, as in the erect posture, by which the integuments are put upon the stretch, and pressure is thus exerted over the part. Some patients are unable to maintain the erect posture without resting the foot of the side affected on a stool, so as to keep the thigh more or less bent upon the pelvis, whereby the integuments, &c., are relaxed. Radiating from the ovarian region, the pains are felt across the loins; they descend towards the thighs and fundament, and are of a dull, dragging, heavy, and sometimes of an overwhelming nature. They are distinguished by the patient from other pains resembling colic, and which depend on uterine contractions, although both species of pain may be experienced at the same time; they are likewise to be distinguished from those *superficial* pains which are caused by reflex nervous action, and which so frequently accompany every species of disorder of the organs of generation. They are, however, seldom so acute as to induce the patient to seek for advice. She may submit to them for years, but should she find them so wearisome to mind and body as to be led to seek advice upon her case, she is frequently treated for uterine disease. This is owing to the opinion, adopted by Hippocrates, and still too implicitly believed, that the uterus is the principal organ of the generative system, and that to the morbid condition of this organ are to be attributed almost all the sufferings of women. Should the patient be married, connexion awakens and renders more or less acute the pains we have described. Ocular inspection, and an attentive manual examination, however, will, in some instances, prove that it is not painful when touched, nor does it present much appearance of disease. In sub-acute ovaritis, the hands placed on the iliac regions can sometimes detect an increase of heat; but these symptoms of ovarian inflammation are overlooked, or attributed to disease of the womb, inflammation of its neck, or to that scape-goat of uterine pathology, only known in England, and called irritable uterus—a disease regarded as neuralgia by some, as a form of dysmenorrhœa by others, and which, having the same symptoms as sub-acute ovaritis, we suppose sometimes to be one of the legionic names of that disease. The late Dr. Ingleby noticed that the descent of the ovaries on the vagina produced in one of his patients all the symptoms of the disease called irritable uterus.

The author then proceeds to show how amenorrhœa, dysmenorrhœa, menorrhagia and hysteria, may be traced to this pathological condition of the ovaries, and sterility and uterine inflammation be secondarily established. We wish that time were allowed us to lay before our readers an extended analysis of this excellent work. We never regret our circumscribed limits so much as upon occasions like the present; but if what we have presented shall induce any to procure this book, and read for themselves, we promise them that they will find material for thought that may dispel much of the obscurity with which this subject is invested.

Indeed the whole subject of ovarian physiology and pathology is, in the language of Dr. Laycock, "too interesting to require any other introduction to the world than its own merits. In support of this proposition, the philanthropist might observe, that all the best feelings of humanity should urge us to continued effort for the welfare of the sex; the political economist might advance, that the power of a people is indissolubly connected with the physical well-being of its females."* In the reproductive system, the ovaria are the all-important constituents; upon them depend the sexual characteristics, and from their pathological condition springs a train of ills too often as little understood as the physiological condition upon which they are based.

The work of Dr. Tilt ends with the following *conclusion*, expressing the deductions to be derived from the preceding pages.

'How does inflammation, by reacting on the ovaries, produce diseases of menstruation?' This was the last of our introductory questions, and if the reader has carefully followed us in our attempts to solve it, he will have seen that in studying the influence of inflammation on the ovaries, and in describing its peculiar characteristics, we have unavoidably detailed those groups of symptoms generally known as *diseases of menstruation.* We think we have successfully shown that these diseases are often the consequences of structural lesions of the ovaries, being in some instances the immediate result of such structural lesions, while in others, sub-acute ovaritis produces diseases of menstruation by the *induction* of organic lesions in the neck of the womb.

We think the following practical deductions, from our previous enquiries, express some truths respecting diseases which are as frequent as they have been hitherto little understood.

1. That amenorrhœa is often the result of sub-acute ovaritis, sometimes the result of the uterine engorgement which it determines.

2. That dysmenorrhœa is often the result of morbid ovulation, and often a symptom of ovarian peritonitis. That frequently sub-acute ova-

* A treatise on the nervous diseases of women, comprising an inquiry into the nature, causes and treatment of spinal and hysterical disorders, by Thomas Laycock, M. D.

ritis, by determining the inflammation and swelling of the neck of the womb is a mediate cause of dysmenorrhœa: the painful symptoms being, in many instances, produced by the partial closure of the neck of the womb, and the consequent effusion of menstrual secretion into the peritonæum.

3. Acute ovaritis, which, by some unexplained process, disposes the engorged uterus to let the vital fluid run to waste.

4. That sub-acute ovaritis, by inducing cerebro-spinal reflex action, in certain pre-disposed subjects, is the most probable cause of hysteria.

We think we have given a sufficient number of cases to illustrate our views. To corroborate them further, would have required a greater space than could be conveniently comprised within the limits of one volume; but if there be truth in what we have advanced, sufficient has been said to put more able observers on the right track; and if we are wrong, the sooner we conclude the better.

We cordially recommend this little work to our readers.

---

*History of Medical Education and Institutions in the United States, from the first settlement of the British Colonies to the year* 1850; *with a chapter on the Present Condition and Wants of the Profession, and the Means necessary for supplying these Wants, and elevating the character and extending the usefulness of the whole Profession.* By N. S. Davis, M. D., Prof. of the Principles and Practice of Medicine in Rush Medical College, &c. &c. &c. Chicago, 1851.

The last chapter of this book, like the postscript of a lady's letter, affords the key to the object with which it was written. We were at a loss for the motive which could have induced the publication of so very dull a réchauffé, (deficient even in the cardinal merit of accuracy,) till some concluding allusions to an institution mentioned in the title page, served to point the moral which we sought, and developed a very ingenious *Annual Announcement* of the "Rush Medical College, located at Chicago, Illinois." With, however, this indirect advertisement of the college, illustrated by the services of Dr. N. S. Davis, in the chair of Principles and Practice of Medicine, we have little concern. But we feel called upon to expose and condemn a very discreditable reduction of the lecture fees, which has been adopted by the Professors of the institution in question.

It appears that the faculty of the Rush Medical College have lowered the expenses of a full course, of seven lectures, to the gross amount of thirty-five dollars; and Dr. N. S. Davis advocates, or rather threatens a further reduction to twenty-five dollars. The obvious purpose of this proceeding is to underbid the neighboring schools. And a degrading competition is invited, (although we trust the influence of such an institution must be powerless,) fatal to the dignity and efficiency of medical instruction.

We need hardly waste an argument against this most pitiful violation of professional propriety. That its only effect will be to consign the originators of the scheme to merited obscurity, we have every confidence. For we believe that the students will estimate these gentlemen at the value they set upon themselves. But we think it our duty to notice the first movement—no matter how insignificant the quarter in which it may appear—in a course which must drive men of talents and attainments from the control of medical education. The subject will, we are glad to see, be brought before the next meeting of the National Medical Association, which we have no doubt will express itself decidedly in the matter.

---

*Operative Surgery.* By Frederick C. Skey, F. R. S. Philadelphia. Blanchard & Lea, 1851. pp. 661.

There is certainly no branch, at least of surgical knowledge, in which we have been more abundantly favored in this country with works of all shapes, sizes and pretensions, in each of the three leading European tongues, than this of operative surgery. It seems to be the alpha and omega of many of the greater as well as the lesser lights of the great chirurgical galaxy. It has too often proved the last as well as the first theme of professional inspiration on both sides of the Atlantic. Indeed, more than one instance might be named among French authorities of note, in which it has been literally the standing labor of love throughout a life time, and yet proved to be only in the course of progress at the close of a career which left it to be carried on, often in the same snail's pace, by some equally voluminous but longer-lived apostle. In view, then, of this ample previous display of skill and learning, we may well pause to enquire into the claims of one who comes to us unheralded by any flourish of trumpets beyond what is sounded by himself, and supported only by the prestige of his own name and station as an "F. R. S."

It is fair, however, to remember as a matter of course that Mr. Skey has had no part or lot in the reprint now before us; and that he intended solely to address those to whom his high standing as a London practitioner and a member of the Council of the Royal College of Surgeons, as well as assistant surgeon of St. Bartholomew's, had long made him favorably known. In this connection we feel bound to say, that the author has not had the chance which we think should be given him in a reprint; for although the letter press appears to be faithful, and the book generally to have been well gotten up, the illustrations are decidedly inferior to those of the original. In this important particular, at any rate, Mr. Skey as well as his American readers, have strong reason to complain. Without intending in this place to agitate the old vexed and sorely vexing question of rights of authorship, we must say that where an author is "obliged to volunteer," he should at least be presented in the garb in which he had himself chosen to appear. But in thus striking at its shadow we are losing sight of the work itself.

It "was undertaken," says the author, "in compliance with the advice of some professional friends, who equally felt with myself the want of a book on the subject of operative surgery, which might become, not simply a guide to the actual operation, and embrace the practical rules required to justify the appeal to the knife, but would embody, at the same time, such principles as should constitute a permanent guide to the practitioner of Operative Surgery, and without which, all claim to its scientific character is lost."

In these words are unfurled the standard of the book; not very clearly it is true, but still sufficiently so to lead us to look with great satisfaction, not to say confidence, for a treatise embodying principles not less than practice, and establishing the art and mystery of surgery on a higher and broader platform than that of the most skilful employment of mere instruments and apparatus.

"The operating surgeon is not a mechanic, but the agent through whose instrumentality are carried into action the highest principles of scientific medicine, principles demanding an intimacy with the soundest physiology. He wields a power more grand, and more critical, and at the same time, more terrible to humanity than the member of any other branch of our profession." "There is no department of medical practice," continues he, "more attractive to the younger members of our profession than that of operative surgery. Its brilliancy, its eclat, its critical influence in the disease, all contribute to this popularity," &c. "Is not this enthusiasm somewhat misapplied? Between the claims to respect of curative and operative surgery, there is no comparison. I venture to say, with all respect to my many superiors in surgical acquirements, that it is the duty of the teacher to expatiate with all the power of his authority on the superiority of the one over the other." Still our author does not seemed disposed to undervalue his operative branch. His great aim, we are informed, is "to fix it on its proper basis," to warn the surgeon "from paths of danger and difficulty" and to test "the question of every operation, *in foro conscientiæ*, before it is undertaken." He has "endeavored, as an English metropolitan surgeon, to carry into execution at least one primary object, viz., to strip the science of Operative Surgery of a false glare," "to check a spirit of reckless experiment, and to repress rather than encourage the resort to the knife as a radical agent."

These passages evince a spirit and design which we cannot too earnestly commend, and we quote them in order to show that the author belongs to the true school, whatever he may be in scholarship, or as a master. Nothing is intimated in the title page, and very little in the preface, of the opportunities and position which are to give Mr. Skey's teaching the necessary weight, but we are soon enlightened on this question in our perusal of the succeeding chapters. The names of Abernethy, Astley Cooper, Lawrence, Brodie, Stanley and others little less distinguished, repeatedly occur as those of his associates in public and private practice; and from one remark we learn that he has enjoyed the advantages of twenty-five years of experience in the practice of St. Bartholomew's Hospital; while in another place we are told that his opportunities have been "as extensive as fall to the lot of most men."

Such an imposing exhibition of his claims together with the fact of his distinction as member of the Council, and more recently

Hunterian orator, of the Royal College of Surgeons, had disposed us to look for at least a respectable fulfilment of the flattering promises of his preface, notwithstanding a damper at its close, in which he vouchsafes the information that the character of his mind "is not attuned to authority, and it has been my practice, no less than my principle in life, to think for myself." But these exalted expectations were not destined to be realized. Disappointment from one end of the book to the other, will well excuse the language of one of his own countrymen, who complains that "instead of new ideas or clear descriptions, which might be recommended to the attention of our readers, we were to meet with nothing better than unmeaning trivialities, interspersed with old exploded notions, and imperfect views of modern improvements."

We do not pretend to assert that there is not a great deal that is very good, and more or less that is actually new, but we seriously doubt whether there is enough of sterling value in the whole to counterbalance the evil influence of what is worse than useless, still less to justify the publication and re-publication of an octavo volume of six hundred and sixty-one closely printed pages.

If it be not the complete and elevated work on the principles and practice of operative surgery in the present state of the science, as we are led to expect by the high sounding professions of the preface, we have a right to take it for some sort of exponent of the precepts and practice now in vogue in the wards of St. Bartholomew, if not in the English metropolis itself. Although Mr. Skey does not "profess to give the multitudinous opinions of other men," he "has not withheld them." "I have quoted," says he, "the opinions entertained by most of the eminent members of the surgical profession, *so far as a general intercourse and an extensive acquaintance have enabled me to command it.*"* And yet he has enjoyed the rare benefit of twenty-five years of experience in St. Bartholomew's hospital, and of practice in the great city of London along with the first surgeons of the age, and in close proximity to those of Dublin, Edinburgh, and of Paris, not to mention the not less renowned practitioners of other parts

*The italics are our own.

of Europe and of this country, as well as throughout the provinces of the United Kingdom! The idle excuse that his mind is "not attuned to authority," affords but paltry satisfaction to the disappointed student who has sought his work in the vain hope of learning, from the very highest sources, a great deal of the true philosophy of practical science—a philosophy untrammeled by authority indeed, but none the less broadly established on the only true and sure foundation of enlarged experience, and equally enlarged and discriminating study of the experience of others. There is much in Mr. Skey's book that is better told and illustrated in books of far humbler pretensions, and prepared by younger men; and we regret to say that, considering all the circumstances of its preparation, there is comparatively little else that is not almost as objectionable as it is peculiar.

In the course of the first two chapters, which are devoted to general observations and the management of wounds, we find, among other things, the statement of the fact that the records of St. Bartholomew's Hospital point to the successful administration of anæsthetic agents in upwards of nine thousand cases; "in not one of which, including the aged and the young, the healthy and infirm, and the asthmatic, has its employment left a stain on its character as an innocuous agent for good." This is strong evidence in its favor, and is not without an equally decided effect on the author's practice, as shown in his constant recommendation of it in nearly all the operations described, and especially the most painful, such as operations on dead bone, for the reduction of dislocation and other similarly painful and tedious in character, or requiring rest and relaxation. The agent he employs is chloroform, gradually administered with free access of fresh air as a diluent during inhalation, the cambric handkerchief being used in preference to the metall c inhalers.

Passing over some peculiar views in regard to the employment of blunt edges and silver knives in certain operations, as well as other 'notions' still more singular in regard to sutures on the face, we come to chapter third, on Dislocations. This whole subject is dispatched in some sixty-five pages. His advice on this topic, in the main, is such as would be approved of here, but his precept in regard to old luxations, does not appear to be either precise or safe according to the prevailing doctrines in this country. He admits, for instance, an increase of difficulty with the protraction of the term of dislocation but does not consider any serious obstacle to be presented "even though many days or even weeks have expired since the occurrence of the accident." He would not limit the time for the attempt at reduction, "unless we carry the question at once into a period of nine months or a year or more." This is, to say the least of it, a very elastic and uncertain kind of rule. Nor is he disposed to regard as important the risk of lacerating the new formed adhesions around a displaced joint. He cannot understand the danger in such cases, and recommends, that "to effect the complete laceration of these adhesions the limb should be subjected, under the influence of chloroform, to powerful extension, and should be forcibly rotated in all directions." According to his account "the immense experience of Sir Astley Cooper has failed to furnish a single case of serious injury consequent on the attempt to reduce any form of dislocation." If our author had taken the trouble to consult a few of the "multitudinous opinions of other men," a little further from his immediate neighborhood, he might have met with more than one authenticated instance of serious results that would teach a striking lesson of prudence in this matter of long standing dislocation.

Bandages and fractures are hastily despatched in two short chapters, and then followed by a pretty full consideration of aneurisms and the treatment of wounded and diseased blood vessels. In this portion of his work he has lost a capital opportunity to bring his book to the present state of progress, by a full discussion of the treatment by compression. We would have been glad to hear something of practical results, so far as they had been observed in the wards of St. Bartholomew, if no where else; and, at all events, we might have been favored with an exhibition of the apparatus most approved by Bellingham and others, together with some sort of appreciation of its value. He appears to know no more about this vitally important question than every well-informed surgeon could tell us four or five years ago, and presents us with a figure of a "tourniquet" that has long since given place to better things among those who are familiar with the improvements of the day.

Chapter 8th gives a very brief view of varicose veins, and their treatment. It is useful in spite of its meagreness, on account of the operative procedure recommended, and justly, as we think, in preference to all others. Every one is aware of the uncertainty as well as great danger of the different cutting and deligating operations for the removal of this distressing infirmity.—"But," says Mr. Skey, "no danger attaches to the destruction of the saphena and its branches, by means of escharotics, which may be employed in the form of caustic issues with all confidence for the removal of varicose veins of the leg. Having tested this practice for many years, and adopted it largely in the out-patient ward of St. Bartholomew's Hospital, and also repeatedly in private practice, I can unhesitatingly say that the treatment is both efficient and safe." We are disposed, on the strength of some experience, to agree with him fully in his estimate of the superior advantages, on every account, of the caustic treatment of varicose veins. We have seen it extensively employed in this city and elsewhere, and always with more satisfactory results than we have been able to discover in the use of any other therapeutic means.

Next in order comes an interesting, but not in any way remarkable chapter on Amputation. Of this, it will suffice to say that the circular is preferred to the flap operation, although the flap operations are duly considered and described. Following Amputations we find a succession of short articles on various topics of greater or less interest, treated of without much regard to order or connection.

Chapter 15th is occupied with hernia, and presents us with as useful a history of the most important points as could be expected in the compass of twenty-nine octavo pages.

In chapters 16 and 17, respectively, we meet with a very cursory account of operations on the external organs of generation in the male, and operations on the male perineum and about the anus. After these, comes a somewhat elaborate discussion and description of the "operations of lithotomy and lithotrity." He takes "lithotrity as the rule, and the cutting operation as the exception;" and in selecting a case suitable in all its bearings for the former, he would require the following conditions: "First well developed manhood. Second, a healthy and readily dilatable urethra. Third, a bladder free from irritation, and capable of retaining at least six ounces of urine ; a condition which infers the absence of prostatic disease. Fourth, a tonic condition of the nervous system ; and Fifth, the presence of a stone of such dimensions as to be readily embraced by the screw of the lithotrite." What is meant precisely, by the screw of the lithotrite we are at a loss to understand ; for although the operation described and advocated is evidently the crushing one, and the instrument therefore lithotriptic, we are still in the dark as to whether in reality the instrument he uses is not as obsolete as the name he gives it. His history of the different instruments employed in the cutting operation is much more full and satisfactory. He advocates the completion of the opening into the bladder with the knife alone.

Lateral curvature is the next topic chosen to enlarge upon, and one which appears to have been a favorite with our author. His suggestions in relation to its treatment are interesting and instructive.

Four chapters follow on a variety of subjects of greater or less importance, including one on the Cæsarian Section and Ovariotomy. These are succeeded by four others upon the different operations on the eye, which, with a useful index to instruments and apparatus required for special operations, terminate the volume.

A pretty thorough examination of the twenty-eight chapters, briefly analyzed above, has brought many matters of record and opinion to our notice, which might be worth especial mention, either pro or con. But we feel under no obligations to the author, to occupy the attention of our readers any longer with such an unattractive and unprofitable topic,—and while we have considered it our sacred duty conscientiously to warn the American practitioner against the influence which the known position of the writer must inevitably secure to him in many places, we must express our unfeigned regret at the necessity which has obliged us to receive in such a manner, one to whom otherwise we would have been delighted to extend a grateful welcome.

*Report of the Pennsylvania Hospital for the Insane, for the year* 1850. By THOMAS S. KIRKBRIDE, M. D., Physician to the Institution. Philadelphia, 1851.

Dr. Kirkbride very justly observes, in the Report before us, that "the general circulation of Hospital reports, containing the results of judicious treatment, has done more to enlighten the public mind in reference to insanity, to stimulate and give proper direction to the efforts of philanthropists, and eventually lead to a liberal provision for the wants of the insane generally, than all other means combined." Of the value of his own contributions during the ten years the Pennsylvania Hospital for the Insane has been in operation under his care, it is hardly necessary for us to speak. They are identified with the history of the subject, and are appreciated both abroad and at home.

The present report, which includes a period of ten years, presents a more extended analysis of the statistical tables collected in the Institution, than Dr. Kirkbride has heretofore attempted. The various facts connected with the causes and treatment of insanity, as afforded by the Institution, have been grouped under fourteen heads. They show:—1. The number and sex of the admissions and discharges. 2. The ages of the patients. 3. The occupations of the male patients. 4. The occupations of of the female patients. 5. The number of single, married, widowers, and widows. 6. The places of nativity of the patients. 7. Their residences. 8. The supposed causes of their insanity. 9. The ages at which their insanity first appeared. 10. The different forms of their insanity. 11. The duration of the disease at the time of admission. 12. The number of the attacks. 13. The state of the patients who have been discharged or died. 14. The number of admissions, discharges, cures, and deaths, in each month since the opening of the Hospital.

Many results of the highest interest are afforded by these statistics,—very satisfactorily establishing the value of the application of tabulation to the subject of insanity. Indeed, as Dr. Kirkbride judiciously argues, "there seems to be no sound reason why the statistics of insanity may not possess as much certainty as those of most other maladies; and the fact that an undue importance has occasionally been given to them, and erroneous deductions drawn from those having reference to a small number of cases, and for short periods of time, as we all know, has frequently occurred, ought scarcely to be considered a sufficient apology for neglecting to note in this form all the circumstances that inquirers may consider as possessing general interest."

In looking over Dr. Kirkbride's comments on the table showing the supposed causes of insanity, our attention was arrested by his allusions to the influence of opium and tobacco in this respect. The subject of the effects of the use of tobacco having been within a short period noticed in our columns, it will probably interest our readers to learn Dr. Kirkbride's views as regards its agency in producing insanity.

"Two cases in men, and five in women, are reported as caused by the use of opium, and four in men as caused by the use of tobacco. In reference to the influence of these articles in producing insanity in the cases referrred to, there did not seem to be any just ground for doubt. Opium is much more used among females than males, and its effects upon the mind, no less than upon the body, are of a most injurious character. The use of tobacco, which is much more restricted to men, has, in many individuals, a most striking effect on the nervous system, and its general use in the community is productive of more serious results than are commonly supposed. Its excessive use is apt to develop gastric derangement and disorders of the nervous system, and renders active other influences that might have been harmless. In many chronic and recent cases of insanity, the effects of a temporary indulgence in it are so striking as to attract the attention of all who are habitually about the patients. After no inconsiderable amount of experience in reference to this article, I have no hesitation in saying that I have never seen anything more than a temporary annoyance, such as would occur in giving up any confirmed habit, result from its entire discontinuance, and by that course alone the complete re-establishment of impaired health, has often been produced."

In connection with the general arrangements of the Institution, it may be well to mention, for the benefit of such of our readers as are connected with hospitals and other public institutions, that Dr. Kirkbride speaks very decidedly in favor of the use of steam for the purposes of heat and ventilation.

"The fixtures for heating several of the wards of the South Wing by steam, have been steadily in use, and continue to give entire satisfaction."

And he adds :—

"Heating by steam or hot water in connection with a forced ventilation, has now been so fully tested under various circumstances, in our highest latitudes, and its great superiority over every other mode now known, so clearly demonstrated, that hereafter public opinion will hardly justify those to whom is intrusted the high responsibility of providing buildings for the permanent accommodation of large numbers, whether in sickness or health, for adopting any of the very imperfect kinds of fixtures heretofore employed."

The Report presents, in conclusion, some interesting particulars in connection with the Pennsylvania Hospital.

"It is now just about a century since the Pennsylvania Hospital, the pioneer institution for the insane in America, was incorporated by the Provincial Assembly, and opened for the reception of patients. With the exception of the Friends' Asylum at Frankford, established in 1817, and the Insane Department of the Philadelphia Almshouse in Blockley, (which a few years since, for the first time, took rank as a curative establishment,) the Pennsylvania Hospital has been the only institution in the State to which any class of her citizens could resort for the treatment of insanity, and it was strictly the only one which offered relief from this malady without cost to the indigent of Pennsylvania.

From the foundation of the Pennsylvania Hospital in 1751 to the present time, 6062 insane persons have been admitted and treated in its wards; of these more than 1000 were poor, who received every care and attention without charge of any kind, and of whom a large proportion were restored to their families in perfect health, and many others in various states of improvement; the number of this class under treatment being only limited to the income of the Institution."

---

*Thermal Ventilation, and other Sanitary Improvements, applicable to Public Buildings, and recently adopted at the New York Hospital: A Discourse Delivered at the Hospital, February* 8*th*, 1851. By John Watson, M. D.

The New York Hospital having lately undergone various alterations with a view to the introduction of every available sanitary improvement, Dr. Watson has been at the pains to publish a detailed account of them, which will be read with great interest by all whose attention has been specially drawn to the subject. One of the chief improvements introduced into the hospital, is the steam apparatus for the purposes of heat and ventilation, which has been for some time so successfully in use at our Alms-House Hospital and at the Pennsylvania Hospital for the Insane.

*Proceedings of the Medical Association of the State of Alabama, begun and held in the City of Mobile, December* 10—14, 1850. With an Appendix. Mobile, 1851.

Among the published transactions of the various State Medical Associations, the volume lately issued by the Medical Association of the state of Alabama deserves a high place. The physicians of that state were among the first to respond to the suggestion of the National Medical Association, in organizing a State Medical Association, and they have maintained the effort with creditable zeal and ability. The volume before us presents a large number of interesting scientific papers, while the debates and occasional addresses are distinguished by the highest tone in medical politics and ethics. The proceedings comprise a very complete history of the diseases of a considerable portion of the state of Alabama, valuable notices of the medical botany of many counties, with reports of several remarkable and interesting cases We have beside an annual address from Dr. Lopez, and a valedictory address from Dr. Lavender, who successively filled the chair of the Association.

We feel that the thanks of the profession generally, are due to the members of the Alabama Association for the spirited manner in which they have carried out their state organization—particularly to Drs. Lopez, Lavender, Percival, L. H. and W. H. Anderson, D. R. Smith, Welch, Wooten, Mason, and Reese, for the valuable contributions with which they have enrichod the volume of "Proceedings."

